# Three-Dimensional Reduced Graphene Oxide Hybrid Nano-Silver Scaffolds with High Antibacterial Properties

**DOI:** 10.3390/s22207952

**Published:** 2022-10-19

**Authors:** Yueshui Lyu, Yingying Shi, Sen Zhu, Yuan Jia, Chunfeng Tong, Shixiong Liu, Bo Sun, Jingxiang Zhang

**Affiliations:** 1 The First School of Clinical Medicine, Lanzhou University, Lanzhou 730000, China; 2 School of Stomatology, Lanzhou University, Lanzhou 730000, China; 3 School of Life Sciences, Lanzhou University, Lanzhou 730000, China; 4 School of Civil Engineering and Mechanics, Lanzhou University, Lanzhou 730000, China; 5 Northwest Research Institute Co., Ltd. of C.R.E.C., Lanzhou 730000, China

**Keywords:** antibacterial device, Ag nanoparticles, graphene, sewage treatment

## Abstract

In recent years, hazardous wastewater treatment has been a complex and global problem. In this work, by considering the antimicrobial activity of Ag nanoparticles (AgNPs) and reduced graphene oxide (rGO), we constructed an antibacterial device (G-AgNP) with AgNPs conformably deposited onto a 3D scaffold of reduced graphene oxide in situ. The major limitation, which is difficult to recycle, of two-dimensional graphene-silver composite materials in previous studies is improved. Characterization techniques, SEM, TEM, XRD, and XPS, confirmed the synthesis of nanocomposites. Attributed to its larger specific area, more active sites, and synergistic enhancement, the G-AgNP device demonstrated the best bacterial removal capacity, with an antibacterial rate for both *E. coli* and *S. aureus* as high as 100% at quite low AgNP contents. The reported G-AgNP has potential application as a wearable sewage treatment device and for the protection of wearable sensors as a promising sterilizing candidate based on its high and stable antibacterial efficiency.

## 1. Introduction

Hazardous wastewater treatment is a complex issue and a global challenge. Many factors contribute to the reduction in water quality and sustainability, of which pathogenic microorganisms are the most common problem [1]. These microorganisms cause waterborne diseases such as hepatitis, cholera, dysentery, cryptosporidiosis, giardiasis, diarrhea and typhoid, and even cancer [2,3]. The critical issues are not only finding cures for these diseases but also the efficient removal of a large number of pathogenic microorganisms from water [4,5]. To date, many techniques and methods have been used for purifying the wastewater, such as biodegradation, carbon adsorption, evaporation and chemical oxidation [6]. Unfortunately, these methods are expensive, inefficient, single-pollutant type, complex, difficult to maintain and generate secondary environmental damage [7]. Therefore, a green, low-cost and advanced wastewater treatment method is urgently needed [8]. Based on previous research, bismuth sillenite crystals appear to be promising photocatalysts for water treatment, particularly for degrading and reducing organic and inorganic contaminants [9]. However, more efforts are required for disinfecting water from bacteria and viruses. In this regard, nanomaterials with antibacterial properties offer new opportunities [10].

Among the reported metal and metal oxide nanoparticles, Ag nanoparticles (AgNPs) are receiving greater consideration in antibacterial fields [11]. AgNPs exhibit antimicrobial activity against fungi, certain viruses, and antibiotic-resistant bacterial strains by report [12]. With regard to their mechanism of action, AgNPs are reported to cause structural and physiological changes in microbial cell membranes, such as accumulation and changes in permeability and membrane potential and the inhibition of respiratory proteins attached to the cell membrane, disrupting the cell’s homeostasis and ultimately leading to microbial cell death [13,14,15]. However, the aggregation of AgNPs reduces the specific surface area of AgNPs and their antibacterial activity [16]. In addition, AgNPs applied to wastewater treatment agent are difficult to recycle [17]. To address this problem, various inorganic or organic materials were employed as support to enhance the stability of AgNPs [18].

In recent years, graphene-based materials have demonstrated enough outstanding properties such as large specific surface area, strong surface tension and oxidative stress [19] to be considered crucial in future technologies and have attracted attention in a variety of research fields such as physics, material science, chemistry and as materials for synthetic super capacitor devices and sensitive microsensors [20]. In addition, certain studies have revealed that the large specific surface area of graphene can make it an excellent carrier for accommodating inorganic nanoparticles [21]. Moreover, graphene has excellent antibacterial properties and have been used as antibacterial agents [22,23,24]. The antibacterial properties and related mechanisms of graphene materials have been recognized by many scholars, who discovered that an effective way to inactivate bacteria is ensuring direct contact between the sharp edges of the nano-walls and bacteria to damage the cell membrane and change its permeability [25,26]. More importantly, graphene- or graphene oxide-loaded metal nanoparticles can maximize the synergistic effect of antibacterial activity and achieve better properties [27], for example AgNPs. Chen et al. [28] decorated graphene oxide sheets with AgNPs and showed that nanocomposites have better antibacterial activity than AgNPs. Deng et al. reported AgNPs could attach on the surface of GO through physisorption, electrostatic binding and charge-transfer interactions as the reduction of Ag+ took place on the GO surface and showed excellent antibacterial activity [18]. However, previous research showed AgNPs alone showed less inhibition in all three bacterial strains as compared to Ag-GNPs nanocomposites. Among all Ag-GNPs nanocomposites, 50% GNPs-Ag are more towards inhibiting pathogens, i.e., 28.78% (*E. coli*), 31.34% (*S. aureus*) and 30.31% (*P. aeruginosa*) as compared to AgNPs, i.e., 19.78% (*E. coli*), 17.91% (*S. aureus*) and 20.97% (*P. aeruginosa*), respectively [29]. Additionally, these scattered nanocomposites did not exist as three-dimensional entities, so it was difficult to collect the Ag–GO nanocomposite from water after antibacterial processes, which was not conducive to the reuse of antibacterial agent as well as the environmental protection.

In this study, firstly, we prepared 3D reduced graphene oxide aerogel (rGOA) by hydrothermal and freeze-drying. This is the basis for constructing a recycled antibacterial device [30]. Secondly, the 3D antibacterial device (G-AgNP) was obtained by deposition of AgNPs on the 3D rGOA in situ. Staphylococcus aureus (*S. aureus*) and Escherichia coli (*E. coli*) were selected as pathogenic microorganisms to validate the antibacterial performance of the G-AgNP device. The antibacterial mechanisms of this device (G-AgNP) are attributed to the combined effects of sharp graphene edge-induced cutting damage and the silver-enhanced oxidation process of reactive oxygen species (ROS). Finally, the G-AgNP was assembled into a sewage filter device to enable efficient sewage filtration and recycling. As a smart antibacterial material, it can be used as a wearable device to protect humans from harmful water and gas filled with bacteria. Additionally, the device could be used in these wearable sensors to protect them from bacteria. 

## 2. Materials and Methods

### 2.1. Reagents and Instruments

Silver nitrate powder from aladdin (Shanghai, China); aqueous ammonia from FANGZHEGN REAGENT (Tianjing, China); glucose and EDA from Shuangshuang Chemical Co., Ltd. (Yantai, China); LB agar from nitrotetrazolium blue chloride (NBT) from aladdin (Shanghai, China); dimethyl sulfoxide (DMSO) from Zeye industry biological (Shanghai, China); paraformaldehyde from Sigma-Aldrich (Shanghai, China).

### 2.2. Fabrication of the Antibacterial Devices

**Figure 1:**
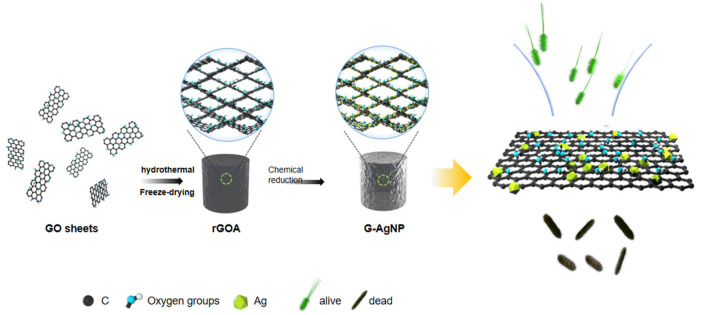
Schematic illustration of the 3D nano-silver-functionalized graphene antibacterial device.

As schematically illustrated in Figure 1 and Supplementary Materials Figure S2, a 3D reduced graphene oxide (3D rGO) scaffold was synthesized through a modified hydrothermal process followed by a freeze-drying process [29]. To obtain 3D reduced graphene oxide (rGOA), the first step was to use a modified Hummer’s process to synthesize graphene oxide (GO) [27]. Then, the GO dispersion was mixed with ethane diamine (EDA) at a volumetric ratio of 5 vol.% and dispersed several times with an ultrasonic homogenizer until the mixture became water like. Next, the solution was added to mold and placed at 120 °C for 6 h. After cooling, the samples were washed with ethyl alcohol and deionized water to remove the residual EDA. Finally, rGOA was obtained after freeze-drying for 24 h.

A 3D nano-silver-functionalized graphene antibacterial device (G-AgNP) was synthesized via an in situ deposition method by the reduction of soluble silver salt under the reaction of both glucose and ammonia. Silver nitrate powder was first weighed precisely to 100, 300 and 500 mg using an electronic analytical balance. Then, the same amount of deionized water was added to the beaker and stirred continuously to clear the sediment. Next, aqueous ammonia was added dropwise to the beaker while stirring with a glass rod. The color of the solution in the beakers turned from colorless to darker brown and back to colorless again, which indicated that ammonia had fully combined with silver ions. The glucose solution, as reduction solution, was made by mixing 0.24 g glucose powder with 12 mL deionized water and then adding equally to each group. The same amount of rGOA was then put into the beakers to soak with solution to fill the pores with ionic liquid, and then they were placed in a suction filter for vacuum drying. Then, cover the sample with plastic film at 80 ° C and heat it for 12 h. After washed by deionized water, samples were removed and freeze-drying for 24 h. Under chemical modification with glucose and ammonia, AgNPs were conformably formed and deposited over the graphene sheets in the microstructure from soluble silver salt as the processor. Finally, G-AgNP was obtained by loading silver onto 3D rGOA. 

### 2.3. Antibacterial Test 

Two kinds of well-described bacterial strains were chosen for evaluation: Escherichia coli (*E. coli* CCTCC AB 93154) and Staphylococcus aureus (*S. aureus* CCTCC AB91093), which were purchased from the China Collection Centre. In brief, microorganisms were picked out and cultured under a sterile in LB liquid medium without agar and then placed into a rotary shaker (200 rpm) at 37 °C for 24 h. The concentration of the microorganism solution was adjusted to 10^7^ CFU/mL for the experiment.

The antibacterial activity of rGOA and G-AgNP was tested using plate counting methods [31]. For the experimental group, after adding a piece of rGOA and G-AgNP antibacterial devices into six-well plate with 2 mL microorganism solution, respectively. Additionally, they were mixed and incubated for 2 h in a constant temperature incubator at 37 °C. Next, 100 μL of bacterial solution after co-culture with rGOA and G-AgNP were diluted in a series of gradient concentrations that were multiplied by 10, and 100 μL of 3 proper concentrations was used to coat the LB agar plate. Three plates were coated with each concentration of bacterial solution and then cultured in a 37 °C constant temperature biochemical incubator for 24 h. The number of colonies in each plate (unit: CFU) was observed and counted. In addition, the control group, which was the bacterial solution without samples, was cultured under the same conditions. As a result, the antibacterial material capacity can be calculated and compared in Equation (1).

(1) $$X=\frac{N-N^{\prime }}{N}\times 100\%$$

where *X* is the antibacterial rate and *N* and *N*’ refer to the number of colonies in the control and experimental groups, respectively.

### 2.4. Intracellular Oxidative Pressure Test Leakage of Cytoplasm

One milliliter of nitrotetrazolium blue chloride (NBT) solution at a concentration of 1 mg/mL was added to the bacterial solution after samples were co-cultured with bacterial solution for 5, 10, 15, 20 and 30 min and further reacted for 30 min at 37 °C. To terminate the reaction, 0.1 mL hydrochloric acid was added to the solution. Then, the suspension was centrifuged for 10 min to collect bacterial cells after removing the supernatant. Next, dimethyl sulfoxide (DMSO) was used to extract NBT inside cells, which was reduced. NBT was diluted to measure absorbance at 575 nm UV light, which could test intracellular oxidative pressure. 

### 2.5. Leakage of Cytoplasm

First, bacterial solutions of the experimental group were filtered with disposable membranes to remove the bacteria inside. Substances inside the cell leak out if the cell membrane breaks, such as DNA and RNA. These substances could be quantitatively analyzed by testing UV absorbance at 260 nm. After that, the optical density (OD) value of the solutions was tested.

### 2.6. Bacterial Fixation and Morphological Observation

*E. coli* in the experimental group and control group that had not been co-cultured with antibacterial samples were collected after culturing for 2 h. After mixing 4% paraformaldehyde with the sediment, bacteria were dehydrated 3 times. Then, they could be observed under an electron microscope (SEM; Hitachi S-4800, Japan) after natural withering. 

### 2.7. Water Filtration Setup and Column Disinfection Performance

To quantify the antibacterial effect of the prepared G-AgNP, *E. coli* and *S. aureus* were used as indicators for bacterial contamination in water for the column experiment. After culturing bacteria, the concentration of bacterial solution was adjusted to 10^7^ CFU/mL. The equipment in this experiment was constructed with a PVC tube, peristaltic pump and columnar G-AgNP filter, which performed the best in the antibacterial test. Place a piece of columnar composite material into a PVC tube, which has a diameter of 1.0 cm and a length of 10 cm. The bottom and top of the column are filled with a layer of glass fiber to fix the material. Then, bacterial solution was pumped from the bottom to the top with the help of a peristaltic pump, which could also be used to adjust the flow velocity of the solution. Next, 100 μL bacterial solution was diluted 3 proper concentrations, coated on LB agar plates, and cultured in a 37 °C incubator for 24 h. Finally, the number of colonies growing on each plate was observed and counted. This method is better visualized through photograph of bacterial growth to determine the antibacterial properties of G-AgNP samples (unit: CFU). 

### 2.8. Microstructural Characterization

The microstructures of the bacteria and device were characterized by scanning electron microscopy (SEM; Hitachi S-4800, Tokyo, Japan). Crystal micrographs of the AgNPs were observed using transmission electron microscopy (TEM; JEM-2100F, Tokyo, Japan). The structures and their chemical components were evaluated by X-ray diffraction (XRD; Rigaku D/MAX-2400, Tokyo, Japan) and X-ray photoelectron spectroscopy (XPS; Thermo Scientific, Waltham, MA, USA).

## 3. Results and Discussion

**Figure 2:**
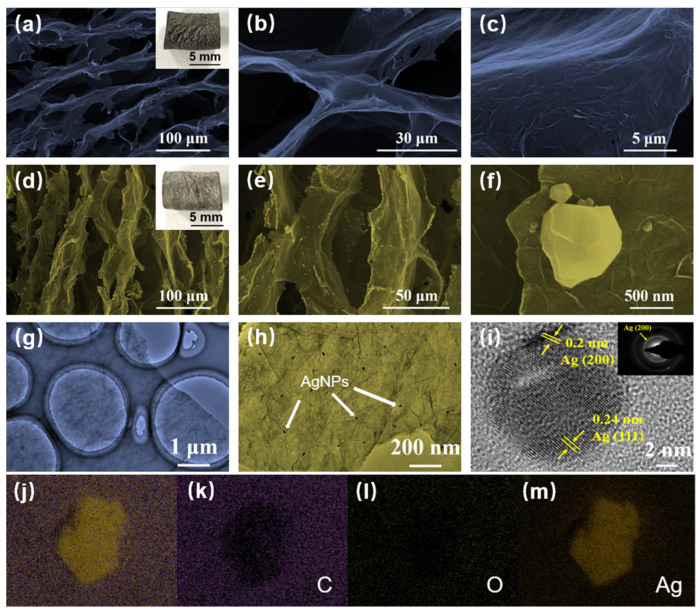
Microstructural characterization. (**a**–**c**) SEM image of 3D graphene scaffolds at different scales. (**d**–**f**) SEM image of the graphene hybrid AgNPs device at different scales. (**g**–**i**) TEM images of the graphene sheet’s wrinkling morphology and AgNPs crystal structure. (**j**–**m**) Element mapping for C, O and Ag.

As shown in Figure 2a–c, the graphene sheets were orderly arranged into honey-comb-like architectures, in which no mineralization particles were present over the wrinkling surfaces. After the in situ deposition reaction, AgNPs were uniformly produced over the graphene sheets at the microscale with an average diameter of several hundred nanometres (100~800 nm) (Figure 2d–f). As presented in Figure 2g–i, the high-resolution TEM observation demonstrates that the AgNPs are uniformly deposited on graphene sheets. The lattice spacings of 0.2 and 0.24 nm corresponding to crystal planes of (200) and (111) [32,33]. Moreover, the element mappings of C, O and Ag for randomly selected locations in Figure 2j directly offer sufficient evidence of the uniform distribution and existence of AgNPs [30]. In Figure 2k–m, the element mappings for C, O and Ag are shown in purple, green and yellow, respectively. As a small smart material, the device is easily made as a wearable device, meanwhile, it can also be assembled in other wearable sensors, thus forming protection to prevent the potential damage of bacteria on wearable sensors.

**Figure 3:**
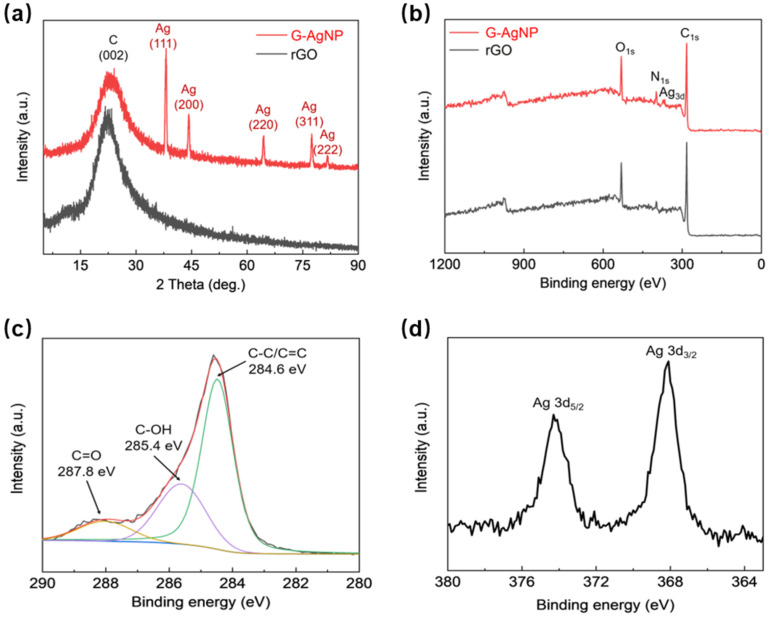
(**a**) XRD investigation. (**b**–**d**) XPS spectra for all elements, C1s and Ag 3d.

XRD and XPS results were obtained to further characterize the chemical composition and structural evolution of the synthesized materials. As shown in Figure 3a, the broad peak at 23.5° corresponds to the C (002) crystal plane, while other sharp peaks at 38.3°, 44.2°, 64.5°, 77.3° and 81.7° are attributed to AgNPs crystal planes of (111), (200), (220), (311) and (222), respectively. According to XPS detections, as shown in Figure 3b, the wide XPS spectrum peaks around 280, 520, and 937 eV was coincident with carbon (C1s), oxygen (O1s), silver (Ag 3d), indicating the existence of 3D graphene scaffolds and nano- silver composites within G-AgNP. Specifically, the deconvolution of C1s spectrum in Figure 3c, gives three typical peaks centered at 287.8, 285.4, 284.6 eV, corresponding to dominate bond within G-AgNP such as C=O, C-OH and C-C/C=C bond, respectively [30]. In addition, the binding energy peaking at approximately 368.4 eV and 374.4 eV represents Ag 3d3/2 and Ag 3d5/2 (Figure 3d) [34]. The deconvolution spectra of C1s and Ag 3d comparatively indicate that AgNPs were physically deposited on the graphene sheet without chemical bonds formed between them.

**Figure 4:**
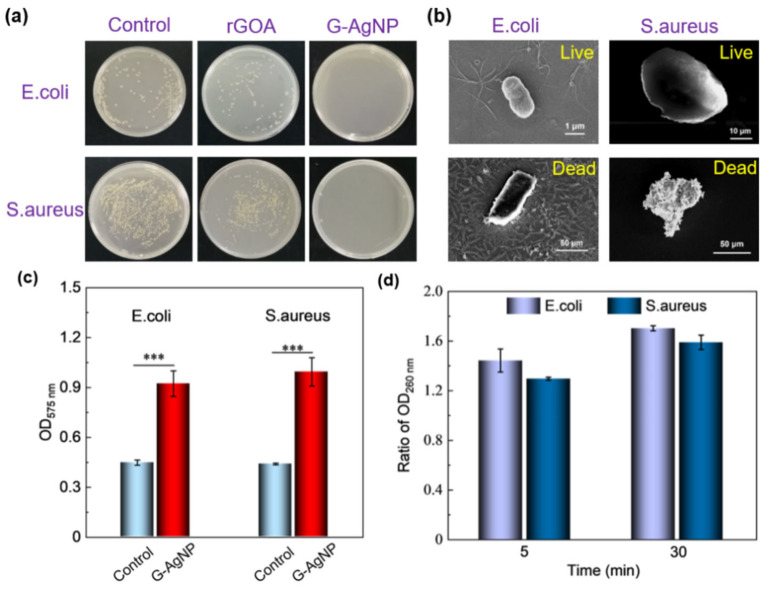
Antibacterial investigation. (**a**) Plates of *E. coli* and *S. aureus* after the antibacterial test. (**b**) The comparative morphologies of *E. coli* and *S. aureus* before and after the antibacterial test. (**c**) The result for NBT test. *** *p* < 0.001. (**d**) The cytoplasm leakage test.

To investigate the antibacterial performance of the G-AgNP device, a qualitative empirical test was conducted to compare the ability of different samples and the control group. Figure 4a presents optical images of antibacterial plates for *E. coli* and *S. aureus* in different groups. As shown in Figure S1, pure graphene-based samples have a certain antibacterial activity with deactivation rates for *E. coli* and *S. aureus* of up to 60.89% and 56.21%, respectively. Because of the synergistic enhancement effect after including AgNPs, the G-AgNP device demonstrates remarkable antibacterial ability with a deactivation rate as large as 100% for even the lowest silver content sample (Ag+ precursor concentration: 3.33 mg/mL).

To understand the AgNPs enhanced antibacterial mechanism, the micromorphology evolution of *E. coli* and *S. aureus* was captured during the experimental process by SEM (Figure 4b). Both of them exhibit structural integrity and smooth cytomembranes before treatment. However, after co-culturing with G-AgNP, the cells become irregularly swollen or disrupted, which is attributed to the increase in the pressure inside the cell induced by ROS production [6]. To further validate the impact of ROS, an NBT test was conducted to measure the pressure inside the cell. Figure 4c shows that the ROS level in the co-culture group was approximately 2 times higher than that in the control group. The difference between the two groups was statistically significant (*p* < 0.001). The cytoplasm generally leaks out after the cell bursts, which can be measured by testing the OD value. As notably depicted in Figure 4d, the OD 260 nm ratio increased from 1.45 to 1.7 for *E. coli* and 1.3 to 1.6 for *S. aureus* at 25 min. This reveals cytomembrane breakage and cytoplasmic leakage under the synergistic interaction of G-AgNP. This validates that the antibacterial mechanism of G-AgNP is related to ROS-induced damage to the cell membrane.

**Figure 5:**
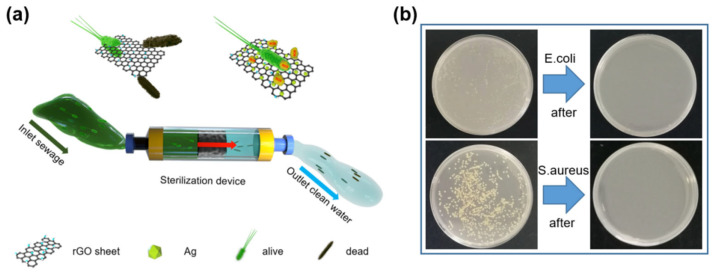
(**a**) Schematic diagram of water filtration setup. (**b**) Column disinfection performance.

To further verify the practicability of the G-AgNP device in real sterilizing applications, as shown in Figure 5a and Figure S3, a functional water filtration device was constructed consisting of a PVC tube, peristaltic pump and columnar G-AgNP filter. The polluted water containing *E. coli* and *S. aureus* was cyclically pumped to flow through the porous G-AgNP. As illustrated in Figure 5b, the water was recovered under abacterial conditions, as all bacteria were removed completely after 5 min of processing, with the antibacterial rate remaining at 100%. As the same time, columnar G-AgNP filter element can also be adjusted, replaced and recycled to avoid secondary contamination of the water. These excellent performance results suggest that the G-AgNP antibacterial device has promising potential for the urgent demand of water treatment.

## 4. Conclusions

In summary, this article has highlighted a AgNP-functionalized 3D graphene antibacterial device, which was synthesized using an in situ deposition process with silver nanoparticles conformably anchored onto a graphene sheet. Attributed to its larger specific area, more active sites, and synergistic enhancement, the G-AgNP device demonstrated the best bacterial removal capacity, with an antibacterial rate for both *E. coli* and *S. aureus* as high as 100% at quite low AgNP contents. The AgNPs enlarged ROS oxidation and sharp edge of the graphene sheet-induced cleavage process were validated as two main contributors to the better antibacterial performance of the hybrid system relative to that the individual constituents of either AgNPs or graphene. At present, we only carry out antibacterial tests on sewage containing two representative pathogens, *E. coli* and *S. aureus*. In the future, we will carry out more extensive research on the application of the G-AgNP device. On the whole, the high efficiency and stable antibacterial properties of the G-AgNP device in sterilization further validated its promising potential as a next-generation candidate for a wearable device to protect humans from harmful water and gas filled with bacteria. Additionally, the device could be used in these wearable sensors to protect them from bacteria.

## Data Availability

The experimental data presented in the present paper are available from the corresponding author upon request.

## Supplementary Materials

The following supporting information can be downloaded at: https://www.mdpi.com/article/10.3390/s22207952/s1, Figure S1: Comparative anti-bacterial tests of (a) The control group, (b) rGOA, and (c) The G-AgNP on agar. Antibacterial killing rate of *E. coli* and *S. aureus* colonies. Figure S2: Different sizes of rGOA can be prepared in large quantities. Figure S3: The G-AgNP filter based anti-bacterial device for polluted water treatment.
