# Supplementary material for: Three-Dimensional Reduced Graphene Oxide Hybrid Nano-Silver Scaffolds with High Antibacterial Properties
Source: Sensors (Basel). 2022 Oct 19;22(20):7952. doi: 10.3390/s22207952 (PMC9607190; doi:10.3390/s22207952)
Supplement: Supplementary file 1 [file sensors-22-07952-s001.zip › sensors-1941934-supplementary.pdf]

Supporting information

Three-dimensional reduced graphene oxide hybrid nano-silver scaffolds with high antibacterial properties

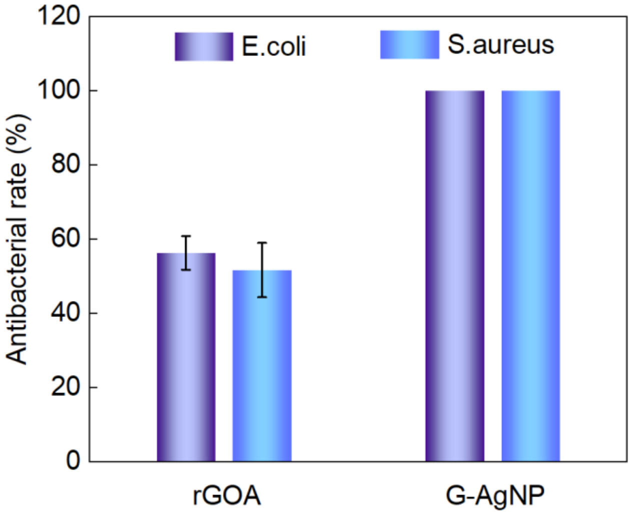

Figure S1. Comparative anti-bacterial tests of (a) The control group, (b) rGOA, and (c) The G-AgNP on agar. Antibacterial killing rate of E. coli and S. aureus colonies.

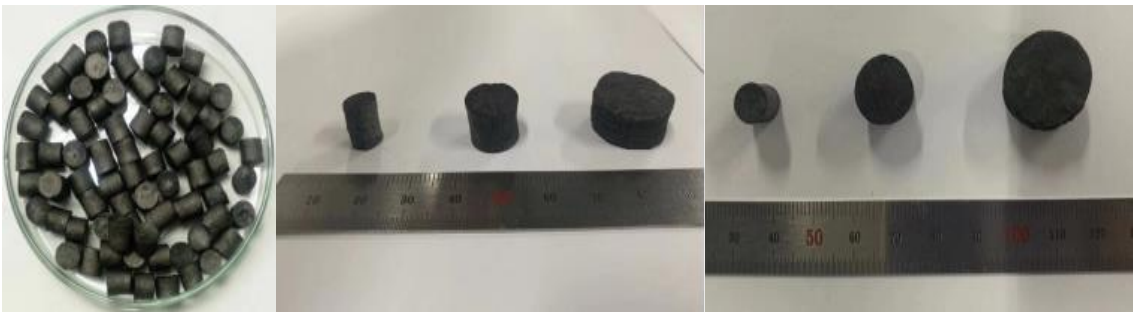

Figure S2. Different sizes of rGOA can be prepared in large quantities.

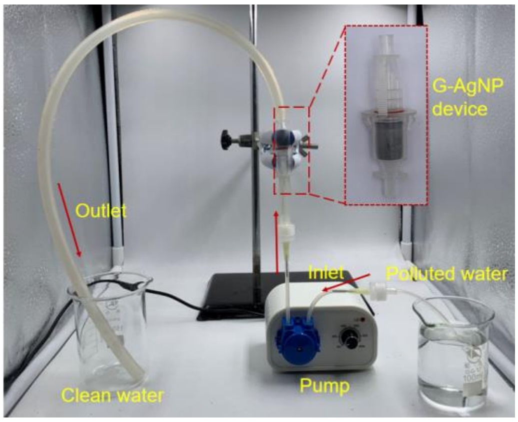

Figure S3. The G-AgNP filter based anti-bacterial device for polluted water treatment.
